# Relationship between the expression of cyclooxygenase 2 and MDR1/P-glycoprotein in invasive breast cancers and their prognostic significance

**DOI:** 10.1186/bcr1313

**Published:** 2005-08-25

**Authors:** Pawel Surowiak, Verena Materna, Rafal Matkowski, Katarzyna Szczuraszek, Jan Kornafel, Andrzej Wojnar, Marek Pudelko, Manfred Dietel, Carsten Denkert, Maciej Zabel, Hermann Lage

**Affiliations:** 1 Institute of Pathology, Charité Campus Mitte, Berlin, Germany; 2 Chair and Department of Histology and Embryology, University School of Medicine, Wrocław, Poland; 3 Lower Silesian Centre of Oncology, Wrocław, Poland; 4 Chair and Department of Oncology, University School of Medicine, Wrocław, Poland; 5 Chair and Department of Histology and Embryology, University School of Medicine, Poznań, Poland

## Abstract

**Introduction:**

Recent reports suggest that expression of the cyclooxygenase 2 (COX-2) enzyme may up-regulate expression of MDR1/P-glycoprotein (MDR1/P-gp), an exponent of resistance to cytostatic drugs. The present study aimed at examining the relationship between the expression of COX-2 and of MDR1/P-gp in a group of breast cancer cases.

**Methods:**

Immunohistochemical reactions were performed using monoclonal antibodies against COX-2 and MDR1/P-gp on samples originating from 104 cases of primary invasive breast cancer.

**Results:**

COX-2-positive cases were shown to demonstrate higher expression of MDR1/P-gp (P < 0.0001). The studies also demonstrate that COX-2 expression was typical for cases of a higher grade (P = 0.01), a shorter overall survival time (P < 0.0001) and a shorter progression-free time (P < 0.0001). In the case of MDR1/P-gp, its higher expression characterised cases of a higher grade (P < 0001), with lymph node involvement (P < 0001), and shorter overall survival (P < 0.0001) and progression-free time (P < 0.0001).

**Conclusion:**

Our studies confirmed the unfavourable prognostic significance of COX-2 and MDR1/P-gp. We also document a relationship between COX-2 and MDR1/P-gp, which suggests that COX-2 inhibitors should be investigated in trials as a treatment supplementary to chemotherapy of breast cancers.

## Introduction

Breast cancer is the most common malignant tumour of females in the western world [1]. The incidence of breast cancer remains high, and its clinical courses are highly variable. It is of general importance to predict the biology of the tumour and, thus, the course of the disease in the individual patient to ensure adequate therapy and patient surveillance [2]. The principal therapeutic approach in breast cancer involves surgery. In advanced cases supplementary therapy is needed, involving pharmacotherapy and/or radiotherapy. Among the pharmacological means, tamoxifen used to be applied most frequently, as well as various chemotherapeutic regimes, including CMF (cyclophosphamide, methothrexate and 5-fluorouracil), anthracyclines and paclitaxel [3,4]. The main reason for therapeutic failure in cases of invasive breast cancers involves resistance to anti-estrogenic treatment and to chemotherapy [5,6]. Identification of the factors that characterise the resistant cases would permit immediate treatment of the patients with alternative therapeutic approaches. These factors could also provide potential targets for studies on novel therapeutic procedures.

Cycloxygenases (COXs) comprise a group of enzymes that participate in the conversion of arachidonic acid to prostaglandins [7]. COX-2 has been characterised as an unfavourable prognostic factor in numerous solid tumours [8-10]. We demonstrated previously in breast cancer patients that expression of COX-2 represents an independent, unfavourable prognostic factor [11]. Numerous *in vivo *and *in vitro *studies indicate that COX-2 inhibitors (coxibs) enhance the efficacy of various anticancer therapy methods [7]. The effect of coxibs on the biology of the tumour has been explained by induction of apoptosis, inhibition of angiogenesis and by a decreased invasive potential of tumour cells [7]. COX-2 has also been shown to up-regulate expression of aromatase [12,13]. In cases of hormone-dependent tumours, such as breast cancer, coxibs might slow down development of the neoplastic disease by decreasing aromatase expression and, therefore, decreasing estrogen secretion. The *in vitro *studies have demonstrated also that COX-2 up-regulates expression of MDR1/P-glycoprotein (MDR1/P-gp) [14], the energy-dependent pump that participates in the phenomenon of multidrug resistance (MDR) [5]. MDR1/P-gp efficiently removes drugs and many commonly used pharmaceuticals from the lipid bilayer. Confirmation of the relationship between COX-2 and MDR1/P-gp in a clinical material may open novel perspectives in the therapy of tumours. Coxibs could be employed as a chemotherapy-supporting treatment, aimed at the inhibition or prevention of the development of the MDR phenomenon.

The present study aimed to examine the relationship between the expression of COX-2 and of MDR1/P-gp in primary invasive breast cancers as well as the definition of their prognostic and predictive values.

## Materials and methods

### Patients

Immunohistochemical analysis was performed retrospectively on tissue samples that were taken for routine diagnostic purposes. The cases were selected based on availability of tissue and were not stratified for known preoperative or pathological prognostic factors. The study was approved by an Institutional Review Board (University School of Medicine, Wrocław, Poland) and the patients gave their informed consent before their inclusion into the study. A total of 104 patients with primary invasive breast cancer who were diagnosed in the years 1993 to 1994 in the Lower Silesian Centre of Oncology in Wrocław, Poland, qualified for the studies. All the patients were subjected to mastectomy and, subsequently treated with radiotherapy and/or chemotherapy and/or hormonotherapy (Table 1). Compliance was monitored by the doctors in charge. The patients were monitored by periodic medical check-ups and ultrasonographic and radiological examinations. During the follow-up period, 23 patients (22%) had recurrent disease and 25 patients (24%) died of the disease. The mean (median) progression-free survival time was 76 months (range 8 to 103 months), while the mean (median) overall survival time was 81 months (range 8 to 103 months).

Fragments sampled from studied tumours were fixed in 10% buffered formaline and embedded in paraffin. In every case, hematoxylin and eosin stained preparations were subjected to histopathological evaluation by two pathologists. The stage of the tumours was assessed according to the TNM classification system [15]. Tumour grade was estimated according to Bloom-Richardson and the modification of Elston and Ellis [16] (Table 1).

### Immunohistochemistry

Freshly cut sections (4 μm) of the formalin-fixed, paraffin embedded tissue were mounted on Superfrost slides (Menzel Gläser, Braunschweig, Germany), dewaxed with xylene, and gradually hydrated. Activity of endogenous peroxidase was blocked by 5 minute exposure to 3% H_2_O_2_. All the studied sections were boiled in Antigen Retrieval Solution (DakoCytomation, Glostrup, Denmark), in the case of COX-2 for 10 minutes and in the case of MDR1/P-gp for 15 minutes. Immunohistochemical reactions were performed using monoclonal mouse antibodies against COX-2 (Cayman Chemical Company, Ann Arbor, MI, USA) at a dilution of 1:2000, monoclonal mouse antibodies (clone C219) against MDR1/P-gp (Alexis Biochemicals, Grünberg, Germany) at a dilution of 1:100 and monoclonal mouse antibodies (clone JSB-1) against MDR1/P-gp (Roche Diagnostics, Mannheim, Germany) at a dilution of 1:100. The antibodies were diluted in Antibody Diluent with background reducing component (DakoCytomation). Tested sections were incubated with antibodies for 1 h at room temperature. Subsequent incubations involved biotinylated antibodies (15 minutes, room temperature) and streptavidin-biotinylated peroxidase complex (15 minutes, room temperature) using a LSAB+ HRP system DakoCytomation). NovaRed (Vector Laboratories, Peterborough, UK) was used as a chromogen (10 minutes, room temperature). All the sections were counterstained with Meyer's hematoxylin.

### Evaluation of reaction intensity

The intensity of immunohistochemical reactions with COX-2 was appraised using a simplified scale. A case was diagnosed as COX-2 positive (1) when expression was observed in all tumour cells or in numerous cell clumps, or as COX-2 negative (0) when no reaction was noted or the reaction was present in only individual tumour cells (<10%). The intensity of immunohistochemical reactions with MDR1/P-gp was appraised using the semi-quantitative immunoreactive score (IRS) scale [17], in which the score reflected both the intensity of the reaction and the proportion of positive cells (Table 2). The final score represented the product of points given for individual characteristics and ranged between 0 and 12. The intensity of immunohistochemical reactions was appraised independently by two pathologists; in doubtful cases, a re-evaluation was performed using a double-headed microscope.

### Control reactions

In each case, control reactions were included, in which specific antibody was substituted by Primary Mouse Negative Control (DakoCytomation). Control reactions were also performed for each of the examined antigens. For MDR1/P-gp, positive controls involved sections of six formalin-fixed and paraffin-embedded human liver samples (from the archive of the Chair and Department of Histology and Embryology, University School of Medicine in Poznañ, Poland) for each antibody. To evaluate specificity of the COX-2 antibody (Cayman Chemical Company) we [18] and other investigators [8] performed blocking experiments using the COX-2 blocking peptide (Cayman Chemical Company) according to the manufacturer's instructions.

### Statistical analysis

Statistical analysis of the results took advantage of Statistica 98 PL software (Statsoft, Krakow, Poland). The employed tests included chi^2 ^test, Spearman's rank correlation and ANOVA rank Kruskal-Wallis test. Kaplan-Meier's statistics and log-rank tests were performed using SPSS software (release 10.0; SPSS Inc., Chicago, IL, USA) to estimate the significance of differences in survival times. The length of survival was defined as the time between the primary surgical treatment and diagnosis of a recurrent tumour or death due to the neoplastic disease. Because the univariate analysis failed to disclose any significant relationships between studied clinicopathological parameters (age, menopausal status, grade and stage) and overall survival and progression free time in studied patients (*P *> 0.05), no multivariate analysis was conducted. The absence of a relationship between the until now most recognised and most effective prognostic factors [19] and patient survival time resulted most probably from the highly uniform character of the examined group of patients. As many as 96% of the examined patients represented the stage II (UICC) [15]. The uniform character of the group allowed a more unequivocal evaluation of the effect of the intensity of expression of the studied protein on the survival time of the patients.

## Results

### Immunostaining in control preparations and in breast cancers

**Figure 1:**
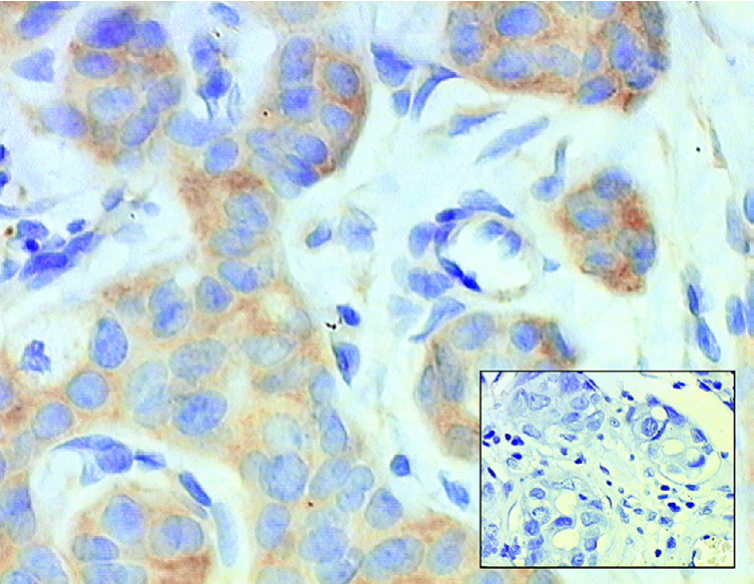
Immunohistochemical localization of cyclooxygenase-2 (red). The inset shows the control reaction with blocking peptide under the same conditions (hematoxylin, ×400).

Immunostaining for COX-2 occurred in a cytoplasmic localization and was of varying intensity among individual cases (Fig. 1). In six COX-2 positive cases, we performed blocking experiments using the COX-2 blocking peptide, as described previously [18]. We found no staining in control preparations (Fig. 1). Expression of COX-2 was noted in 46 cases (44%).

**Figure 2:**
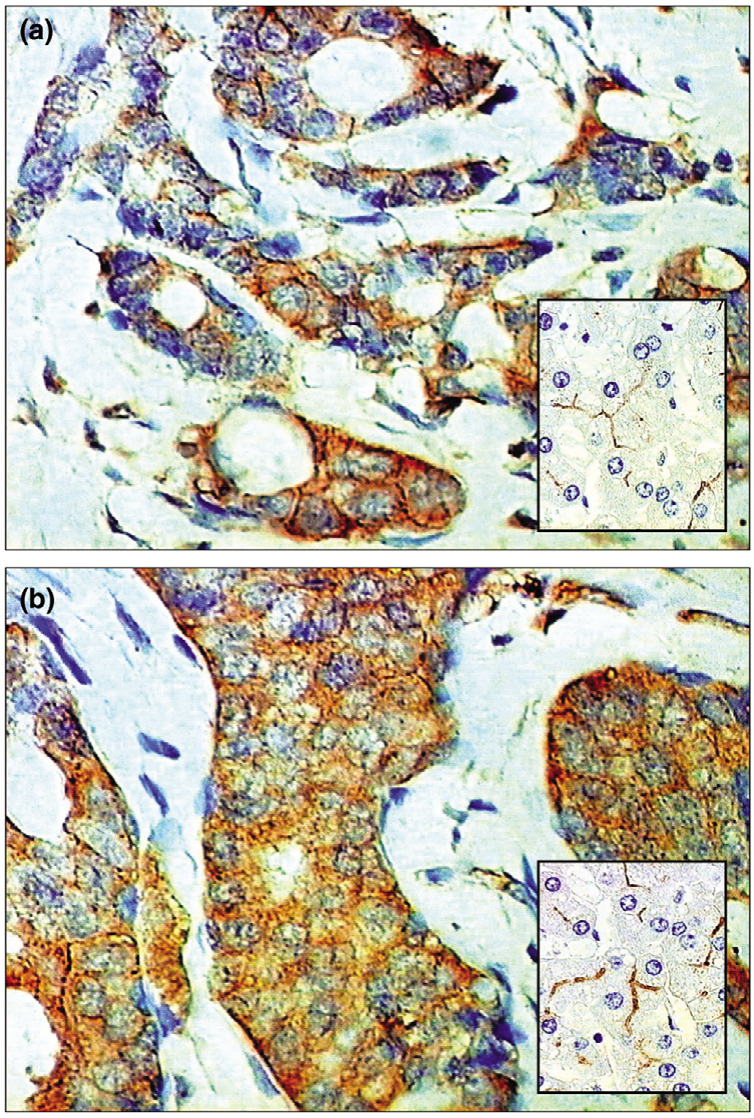
Immunohistochemical localization of MDR1/P-glycoprotein (red). **(a) **With antibody C219 in breast cancer (showing cytoplasmic and membrane localizations in cancer cells) and healthy human liver (inset) (hematoxylin, ×400). **(b) **With antibody JSB-1 in breast cancer (showing cytoplasmic and membrane localizations in cancer cells) and healthy human liver (inset) (hematoxylin, ×400).

Immunostaining for MDR1/P-gp occurred in membranes in samples of healthy human liver and in the cytoplasm and membranes in breast cancers, and was of varying intensity among individual cases (Fig. 2a,b). The mean overall immunoreactivity score for MDR1/P-gp expression detected with antibody C219 was 3.45 ± 3.49 standard deviation (range: 0 IRS to 12 IRS) and with antibody JSB-1 was 3.34 ± 3.49 standard deviation (range: 0 IRS to 12 IRS). We found a strict positive correlation between expression with C219 and JSB-1 (Spearman's rank correlation, R = 0.99, *P *< 0.001).

**Figure 3:**
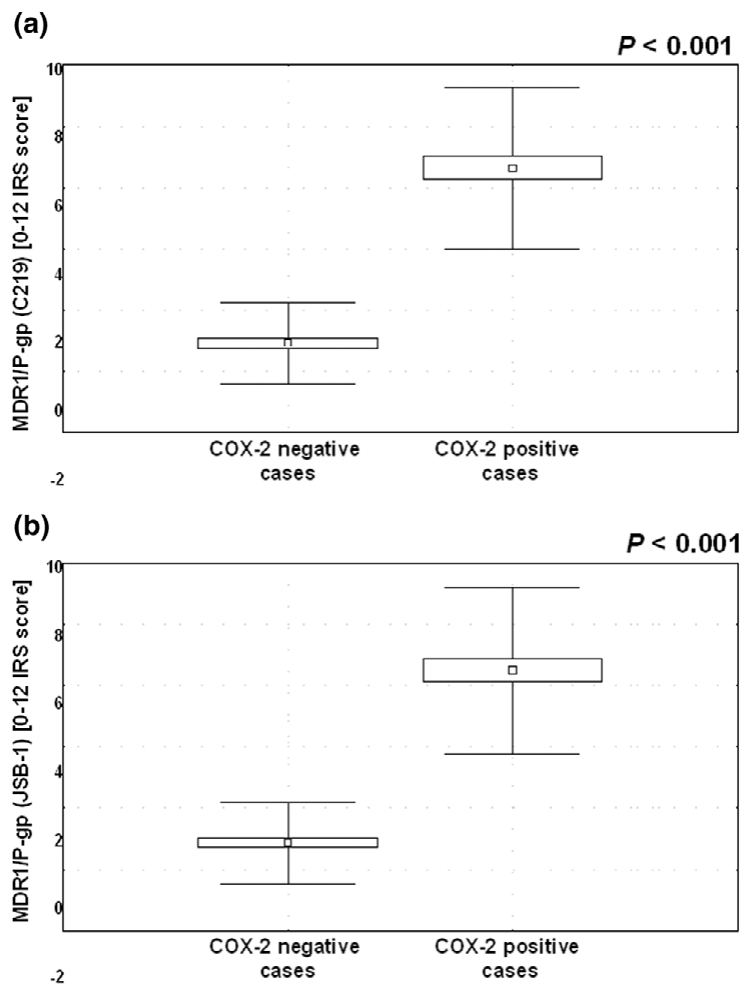
Correlation between cyclooxygenase-2 (COX-2) and MDR1/P-glycoprotein (MDR1/P-gp) expression. **(a) **Correlation between COX-2 and MDR1/P-gp expression detected with antibody C219. **(b) **Correlation between COX-2 and MDR1/P-gp expression detected with antibody JSB-1 in breast cancers. Cases with COX-2 expression show higher MDR1/P-gp expression (ANOVA Kruskall-Wallis rank test, *P *< 0.001).

Using the ANOVA rank Kruskal-Wallis test, we examined the relationship between COX-2 expression and the overall immunoreactivity score of MDR1/P-gp expression. We found that the overall immunoreactivity score of MDR1/P-gp expression with C219 and JSB-1 was significantly higher in cases showing expression of COX-2 (*P *< 0.0001) (Fig. 3a,b; Table 3).

Chi^2 ^tests were also used to analyse the relationships between the intensity of COX-2 and MDR1/P-gp expression on the one hand and the grade, stage, pT (UICC) [15], pN (UICC) [15] and menopausal status of studied patients on the other. We found that at a grade of G3, a significantly higher proportion of cases manifested COX-2 expression compared to patients with a G2 grade (Table 4). The pN1 cases were also shown to exhibit a higher overall immunoreactivity score for C219 and JSB-1 compared to pN0 cases (Table 4); similarly, cases with a G3 grade showed a higher overall immunoreactivity score for C219 and JSB-1 compared to those with a G2 grade (Table 4).

### COX-2 and MDR1/P-gp expression and patient survival

**Figure 4:**
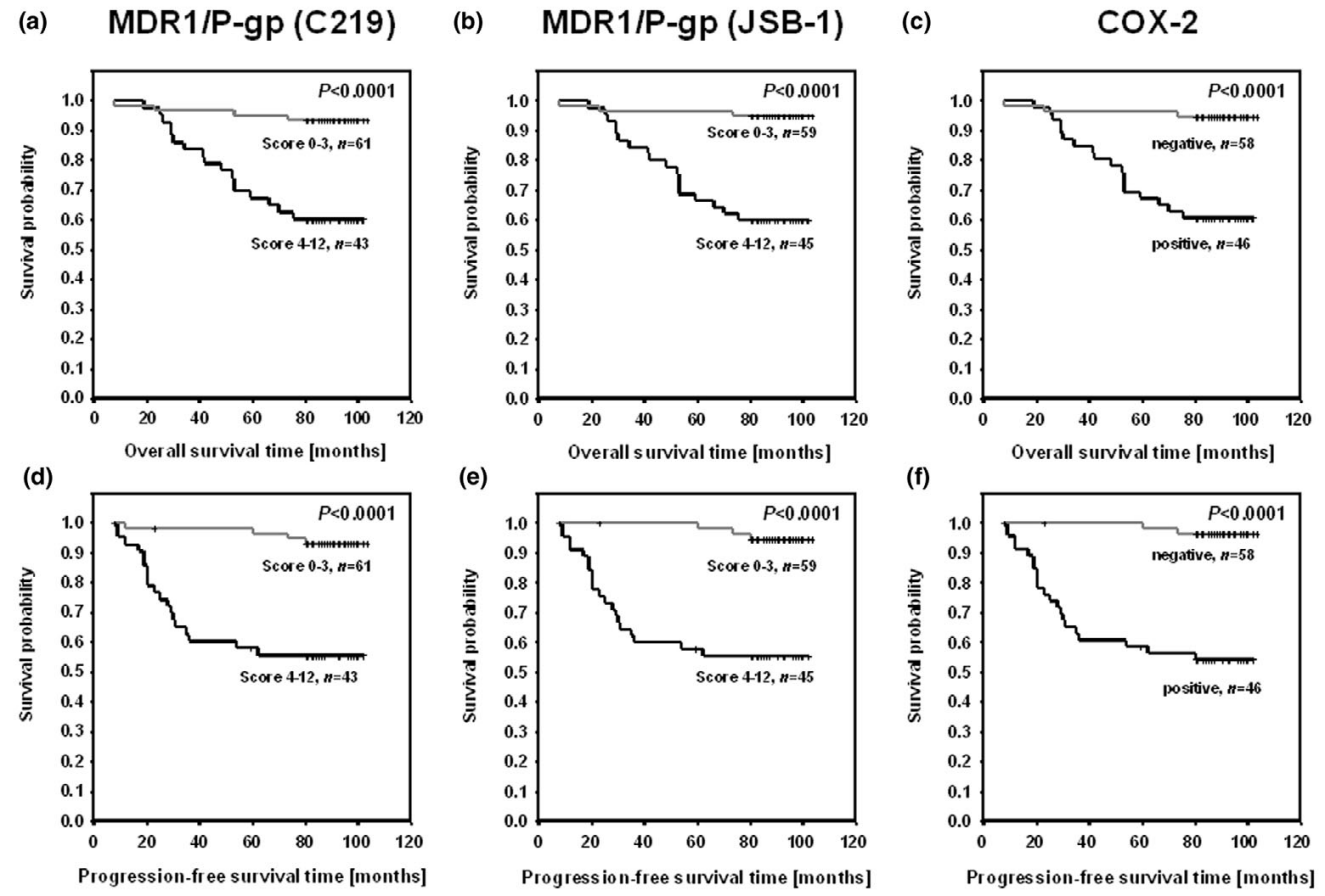
Kaplan-Meier analysis of the complete group of 104 breast cancer patients. **(a) **Patients with a lower overall immunoreactivity score for MDR1/P-glycoprotein (MDR1/P-gp) expression detected with antibody C219 exhibit significantly longer overall survival. **(b) **Patients with a lower overall immunoreactivity score for MDR1/P-gp expression detected with antibody JSB-1 exhibit significantly longer overall survival. **(c) **Cyclooxygenase-2 (COX-2) negative cases exhibit significantly longer overall survival. **(d) **Patients with a lower overall immunoreactivity score for MDR1/P-gp expression detected with antibody C219 exhibit significantly longer progression-free survival. **(e) **Patients with a lower overall immunoreactivity score for MDR1/P-gp expression detected with antibody JSB-1 exhibit significantly longer progression-free survival. **(f) **COX-2 negative cases exhibit significantly longer progression-free survival.

**Figure 5:**
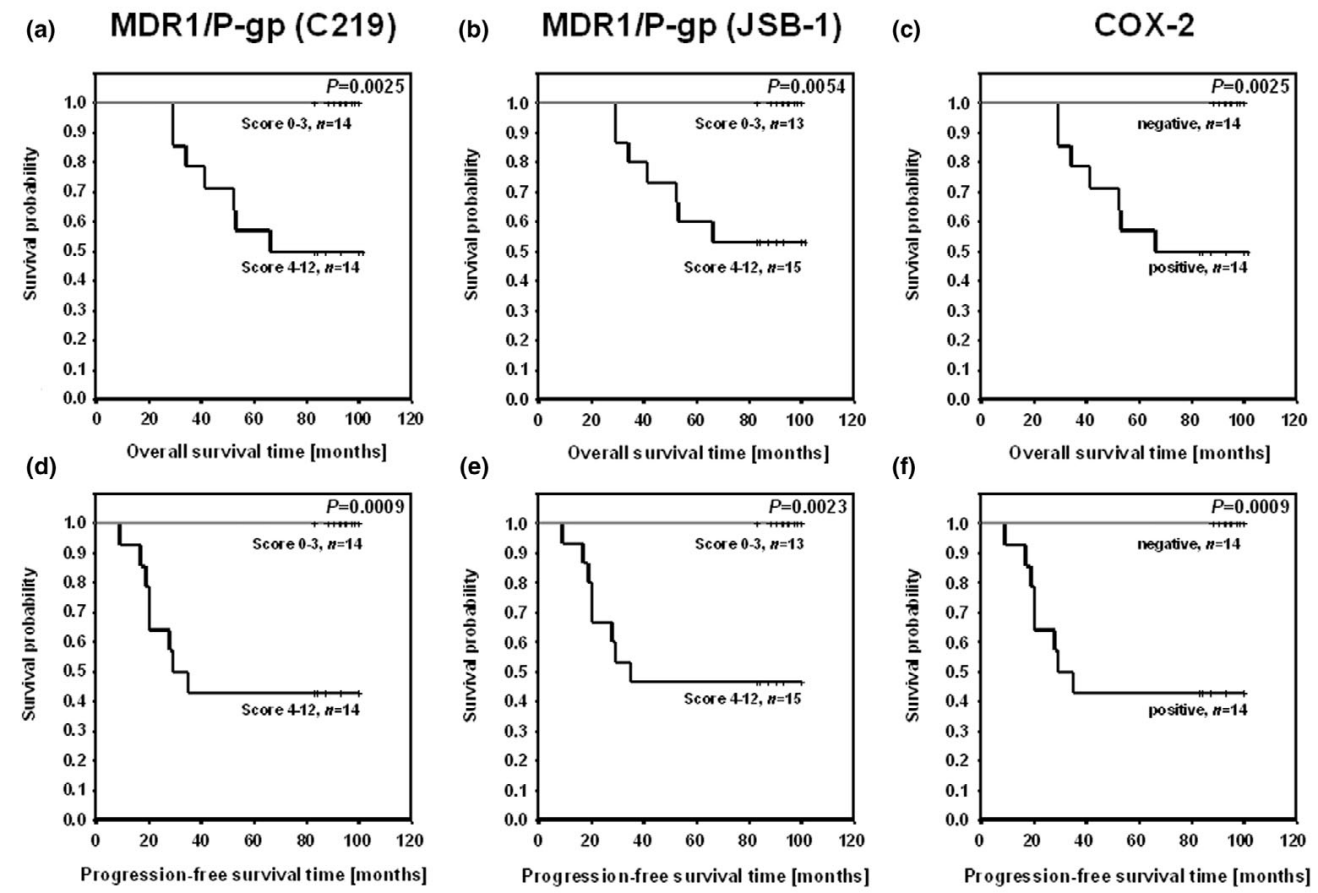
Kaplan-Meier analysis of 28 breast cancer patients treated postoperatively with chemotherapy. **(a) **Patients with a lower overall immunoreactivity score for MDR1/P-glycoprotein (MDR1/P-gp) expression detected with antibody C219 exhibit significantly longer overall survival. **(b) **Patients with a lower overall immunoreactivity score for MDR1/P-gp expression detected with antibody JSB-1 exhibit significantly longer overall survival. **(c) **Cyclooxygenase-2 (COX-2) negative cases exhibit significantly longer overall survival. **(d) **Patients with lower overall immunoreactivity score for MDR1/P-gp expression detected with antibody C219 exhibit significantly longer progression-free survival. **(e) **Patients with lower overall immunoreactivity score for MDR1/P-gp expression detected with antibody JSB-1 exhibit significantly longer progression-free survival. **(f) **COX-2 negative cases exhibit significantly longer progression-free survival.

Kaplan-Meier statistics were used to analyse overall survival time and progression-free survival. In the entire study group, the COX-2 positive cases manifested a significantly shorter overall survival time and progression-free survival compared to COX-2 negative cases (Fig. 4c,f). Cases with an overall C219 and JSB-1 immunoreactivity score between 0 and 3 were also shown to manifest a significantly extended overall survival time and progression-free survival compared to cases with an overall C219 and JSB-1 immunoreactivity score between 4 and 12 (Fig. 4a,b,d,e). The same results were obtained in the subgroup of patients that, following surgery, were treated only with chemotherapy (Fig. 5a–f).

## Discussion

We have described the expression of COX-2 and MDR1/P-gp proteins detected by immunohistochemistry in primary invasive breast cancers. Following the recommendations of the St Judge MDR Workshop on 'Methods to Detect MDR1/P-gp-associated Multidrug Resistance' [20], we have used two different monoclonal antibodies (C219 and JSB-1) directed against MDR1/P-gp. We found a strict positive correlation between MDR1/P-gp expression detected with C219 and JSB-1. We have confirmed that the studied proteins are expressed in a subset of breast cancers [11,21-23]. No relationship was discovered between COX-2 expression and such clinicopathological traits as pT, pN, stage or menopausal status. A higher proportion of COX-2 positive cases was noted in G3 compared to G2 patients. Previously, we noted that COX-2 expression was significantly associated with higher grade, lymph node status and larger tumour size [11]. Ristimäki *et al. *[21] described COX-2 expression using a tissue microarray in 1,576 cases of invasive breast cancer. They demonstrated that elevated COX-2 expression was associated with a large tumour size and high histological grade. In the present study, we have corroborated the positive correlation between COX-2 expression and the unfavourable clinicopathological prognostic indices in another group of patients. We have shown that a higher overall MDR1/P-gp immunoreactivity score is associated with lymph node involvement and a higher histological grade. Numerous authors have demonstrated a positive correlation between MDR1/P-gp expression and tumour stage [24,25]. To our knowledge, this study demonstrates for the first time a significant positive correlation between overall MDR1/P-gp immunoreactivity score and grade of breast cancer tumours. Thus, expression of MDR1/P-gp is typical for the less differentiated cases of breast cancer.

Expression of COX-2 in tumour cells represents an unfavourable prognostic factor in numerous tumours [9,10]. We [11] and other authors [21] have previously demonstrated that COX-2 represents an independent unfavourable prognostic factor in breast cancers. In this study, we have shown that COX-2 positive cases exhibit a significantly shorter overall survival and progression-free time in the entire study group and in a group of patients treated postoperatively with cytostatic drugs. Thus, we have confirmed the previously described observations that COX-2 expression in breast cancer is a negative prognostic factor.

The unfavourable prognostic significance of MDR1/P-gp expression has been documented in several tumours, including breast cancer [24,26-29]. Most of the studies have described the negative prognostic significance in breast cancer cases treated with chemotherapy. Few of the studies have suggested that MDR1/P-gp may also participate in the resistance to hormonal therapy [28]. In our study, we have shown, both in the entire group of patients (in whom surgery was followed by hormonal therapy and/or radiotherapy) and in the group of patients postoperatively treated with chemotherapy, that patients with a higher overall MDR1/P-gp immunoreactivity score exhibited a significantly shorter overall survival and progression-free time. As shown by our data, expression of MDR1/P-gp may not only be linked to resistance to cytostatic drugs but, considering that the higher overall MDR1/P-gp immunoreactivity score is associated with lymph node involvement and higher histological grade, may also represent an unfavourable prognostic factor independent of the applied therapy.

Several reports have suggested that elevated expression of COX-2 may stimulate expression of MDR1/P-gp. Such a phenomenon has been demonstrated, for example, in mesangial cells of rat kidneys, in which transfection with the *COX-2 *gene was followed by an increase in MDR1/P-gp expression [14], and in cells of the gastric mucosa during infection with *Helicobacter pylori *[30]. Ratnasinghe *et al. *[31] have described a positive correlation between COX-2 and MDR1/P-gp expression in breast cancer cases and cell lines. In this study, we confirm that a higher overall MDR1/P-gp immunoreactivity score is typical for COX-2 positive breast cancer cases. To our knowledge, this study demonstrates for the first time a negative prognostic significance of COX-2 and MDR1/P-gp coexpression in breast cancers. The data suggest that clinical studies should be performed on coxibs in chemotherapy-supporting treatment. Apart from their anti-tumour effects, these drugs might prevent the development of, or decrease the intensity of, the already existing MDR phenomenon.

## Conclusion

We have shown the predictive significance of the immunohistochemical estimation of COX-2 and MDR1/P-gp expression in human breast cancers. We found a positive correlation between COX-2 and MDR1/P-gp expression and demonstrated that COX-2 and MDR1/P-gp are unfavourable prognostic factors in breast cancers and unfavourable predictive factors in chemotherapy-treated breast cancer cases. Clinical studies should be performed on coxibs as a supporting treatment in breast cancer chemotherapy.

## Abbreviations

COX = cyclooxygenase; coxibs = cyclooxygenase 2 inhibitors; IRS = immunoreactive score; MDR = multidrug resistance; MDR1/P-gp = MDR1/P-glycoprotein.

## Competing interests

The authors declare that they have no competing interests.

## Authors' contributions

PS contributed to the conception and design of the study, performed immunohistochemistry experiments, statistics and interpretation of data. VM contributed to the conception and design of the study, statistics and interpretation of data. RM performed clinical data analysis and interpretation of data. KS, AW and MP performed immunohistochemistry experiments and contributed to statistical analysis and interpretation of data. JK was involved in clinical data analysis and interpretation of data. MD, CD, HL and MZ contributed to the conception and design of the study and interpretation of data. All the authors read and approved the final manuscript.

## Figures and Tables

**Table 1:** Patient and tumour characteristics

Characteristics	No. (%)
All patients	104 (100)
Age (years; mean 56.2)	
≤ 50	33 (32)
51–60	29 (28)
>60	42 (40)
Menopause	
Premenopausal	30 (29)
Postmenopausal	74 (71)
Grade	
2	71 (68)
3	33 (32)
pT^b^	
1	17 (16)
2	86 (83)
4	1 (1)
pN^b^	
0	29 (28)
1	75 (72)
pM^b^	
0	104 (100)
Stage^b^	
I	3 (3)
IIa	40 (38)
IIb	60 (58)
IIIb	1 (1)
Histology	
Ductal	103 (99)
Scirrhous	1 (1)
Therapy^a^	
Tamoxifen	70 (67)
Radiotherapy	51 (49)
Cyclophosphamide/Methotrexate/5-Fluorouracil	28 (27)
Cyclophosphamide/Adriamycin/5-Fluorouracil	1 (1)
Cyclophosphamide/Adriamycin	1 (1)
Progesterone	1 (1)
Letrozol	1 (1)

^a^Some patients received more than one special treatment.

^b^According to [15]

**Table 2:** Evaluation criteria of MDR1/P-gp expression using the immunoreactive score [17]

Percentage of positive cells	Points	Intensity of reaction	Points
No positive cells	0	No reaction	0
<10%	1	Weak reaction	1
10–50%	2	Moderate reaction	2
51–80%	3	Intense reaction	3
>80%	4		

MDR1/P-gp, MDR1/P-glycoprotein.

**Table 3:** Relationship between COX-2 and MDR1/P-gp (detected with C219 or JSB-1 antibodies) expression (Chi^2^-Test, *P *< 0.001)

	COX-2 positive cases	COX-2 negative cases	Sum
Entire study group (n = 104)			
C219 low [IRS 0–3]	56	5	61
C219 high [IRS 4–12]	2	41	43
Sum	58	46	104
JSB-1 low [IRS 0–3]	55	4	59
JSB-1 high [IRS 4–12]	3	42	45
Sum	58	46	104
Patients postoperatively treated only with chemotherapy (n = 28)			
C219 low [IRS 0–3]	13	1	14
C219 high [IRS 4–12]	1	13	14
Sum	14	14	28
JSB-1 low [IRS 0–3]	12	1	13
JSB-1 high [IRS 4–12]	2	13	15
Sum	14	14	28

COX-2, cyclooxygenase-2; MDR1/P-gp, MDR1/P-glycoprotein; IRS, immunoreactive score [17].

**Table 4:** Relationship between COX-2 and MDR1/P-gp (detected with C219 or JSB-1 antibodies) expression and clinicopathologic factors

Characteristics	No. of patients (%)								
	COX-2 positive	COX-2 negative	*P *value Chi^2 ^test	C219 high (IRS 4–12)	C219 low (IRS 0–3)	*P *value Chi^2 ^test	JSB-1 high (IRS 4–12)	JSB-1 low (IRS 0–3)	*P *value Chi^2 ^test

pT									
1	8 (8)	9 (9)		7 (7)	10 (10)		8 (8)	9 (9)	
2	37 (36)	49 (47)		35 (34)	51 (49)		36 (35)	50 (48)	
4	1 (1)	0 (0)	0.4954	1 (1)	0 (0)	0.7869	1 (1)	0 (0)	0.6306
pN									
0	10 (10)	19 (18)		11 (11)	18 (17)		11 (11)	18 (17)	
1	36 (35)	39 (37)	0.2117	32 (31)	43 (41)	<0.001	34 (33)	41 (39)	<0.001
Stage									
I	1 (1)	2 (2)		1 (1)	2 (2)		1 (1)	2 (2)	
IIa	16 (15)	24 (23)		16 (15)	24 (23)		17 (16)	23 (22)	
IIb	28 (27)	32 (31)		25 (24)	35 (34)		29 (28)	31 (30)	
IIIb	1 (1)	0 (0)	0.6628	1 (1)	0 (0)	0.5915	1 (1)	0 (0)	0.5874
Grade									
2	26 (25)	45 (43)		25 (24)	46 (44)		26 (25)	45 (43)	
3	20 (19)	13 (13)	0.0135	18 (17)	15 (14)	<0.001	19 (18)	14 (13)	< 0.001
Menopause									
Praemenopausal	12 (12)	18 (17)		13 (13)	17 (16)		14 (13)	16 (15)	
Postmenopausal	34 (33)	40 (38)	0.4553	30 (30)	44 (42)	0.8307	31 (30)	43 (41)	0.9703

COX-2, cyclooxygenase-2; MDR1/P-gp, MDR1/P-glycoprotein; IRS, immunoreactive score.
